# Effects of Nintedanib on Quantitative Lung Fibrosis Score in Idiopathic Pulmonary Fibrosis

**DOI:** 10.2174/1874306402014010022

**Published:** 2020-09-22

**Authors:** Lisa Lancaster, Jonathan Goldin, Matthias Trampisch, Grace Hyun Kim, Jonathan Ilowite, Lawrence Homik, David L. Hotchkin, Mitchell Kaye, Christopher J. Ryerson, Nesrin Mogulkoc, Craig S Conoscenti

**Affiliations:** 1 Department of Medicine, Vanderbilt University Medical Center, Nashville, Tennessee, USA; 2 Department of Radiology, University of California, Los Angeles, California, USA; 3 Boehringer Ingelheim International GmbH, Ingelheim am Rhein, Germany; 4 Department of Biostatistics, University of California, Los Angeles, California, USA; 5 Division of Pulmonary and Critical Care Medicine, Department of Medicine, Hofstra North Shore-LIJ School of Medicine, New Hyde Park, New York; 6 Department of Respiratory Medicine and Bronchoscopy, Winnipeg Clinic, Winnipeg, Manitoba, Canada; 7 The Oregon Clinic, Division of Pulmonary, Critical Care & Sleep Medicine, Portland, Oregon, USA; 8 Department of Pulmonary Medicine, Minnesota Lung Center, Ltd., Minneapolis, Minnesota, USA; 9 Department of Medicine & Centre for Heart Lung Innovation, University of British Columbia, Vancouver, Canada; 10 Department of Pulmonology, Ege University Hospital, Bornova, Izmir, Turkey; 11 Boehringer Ingelheim Pharmaceuticals, Inc., Ridgefield, Connecticut, USA

**Keywords:** Disease progression, Tomography, Lung diseases, Interstitial, Exercise test, Therapeutics

## Abstract

**Background:**

Nintedanib slows disease progression in patients with Idiopathic Pulmonary Fibrosis (IPF) by reducing decline in Forced Vital Capacity (FVC). The effects of nintedanib on abnormalities on high-resolution computed tomography scans have not been previously studied.

**Objective:**

We conducted a Phase IIIb trial to assess the effects of nintedanib on changes in Quantitative Lung Fibrosis (QLF) score and other measures of disease progression in patients with IPF.

**Methods:**

113 patients were randomized 1:1 to receive nintedanib 150 mg bid or placebo double-blind for ≥6 months, followed by open-label nintedanib. The primary endpoint was the relative change from baseline in QLF score (%) at month 6. Analyses were descriptive and exploratory.

**Results:**

Adjusted mean relative changes from baseline in QLF score at month 6 were 11.4% in the nintedanib group (n=42) and 14.6% in the placebo group (n=45) (difference 3.2% [95% CI: −9.2, 15.6]). Adjusted mean absolute changes from baseline in QLF score at month 6 were 0.98% and 1.33% in these groups, respectively (difference 0.35% [95% CI: −1.27, 1.96]). Adjusted mean absolute changes from baseline in FVC at month 6 were −14.2 mL and −83.2 mL in the nintedanib (n=54) and placebo (n=54) groups, respectively (difference 69.0 mL [95% CI: −8.7, 146.8]).

**Conclusion:**

Exploratory data suggest that in patients with IPF, 6 months’ treatment with nintedanib was associated with a numerically smaller degree of fibrotic change in the lungs and reduced FVC decline versus placebo. These data support previous findings that nintedanib slows the progression of IPF.

## 1 INTRODUCTION

Idiopathic Pulmonary Fibrosis (IPF) is a progressive fibrosing Interstitial Lung Disease (ILD) [1]. As IPF progresses, lung function declines, dyspnea and cough worsen, exercise capacity is reduced and health-related quality of life deteriorates [2, 3]. Decline in Forced Vital Capacity (FVC) and a reduction in the distance walked over 6 minutes (6-minute walk distance, 6MWD) are predictors of mortality in patients with IPF [4-8].

IPF is characterized by the presence of a Usual Interstitial Pneumonia (UIP) pattern on High-Resolution Computed Tomography (HRCT). A typical UIP pattern includes subpleural basal predominance and honeycombing with or without peripheral traction bronchiectasis or bronchiolectasis [1]. The extent of the lung affected by reticulation and honeycombing, and changes in this extent over time, are predictors of mortality in patients with IPF [9-14]. There is increasing interest in the role of HRCT in assessing disease progression and the efficacy of treatments. Computer algorithms, developed by computer vision and/or deep learning, can detect and quantify the amount of parenchymal lung disease on an HRCT scan. The Quantitative Lung Fibrosis (QLF) score measures the extent of reticular patterns with architectural distortion due to fibrosis using a support vector machine classifier and has been shown to correlate with lung function measurements in patients with ILDs [15-23].

Nintedanib is an approved treatment for IPF. In the Phase II TOMORROW trial [24] and the two Phase III INPULSIS^®^ trials [25], nintedanib 150 mg bid significantly reduced the annual rate of decline in FVC in patients with IPF compared with placebo. Here we describe the results of a Phase IIIb placebo-controlled trial (clinicaltrials.gov, NCT01979952) conducted to assess the effects of nintedanib on changes in QLF score, lung function, exercise capacity and patient-reported outcomes in patients with IPF.

## 2 METHODS

### 2.1 Trial Design

Eligible patients had IPF diagnosed within 5 years, confirmed by the investigator based on the 2011 ATS/ERS/JRS/ALAT guidelines [26], FVC ≥50% predicted and DLco 30–79% predicted. The HRCT scans used for diagnosis were taken <24 months before screening. Patients whose HRCT scan showed a pattern of possible UIP or inconsistent with UIP had confirmatory pathology reviewed centrally. Exclusion criteria included alanine transaminase (ALT), aspartate aminotransferase (AST), or total bilirubin >1.5 × upper limit of normal and bleeding risk (*e.g*., full-dose anticoagulation or high-dose antiplatelet therapy). Patients who required >12 L/min oxygen, were not ambulatory, could not complete a 6MWT, or required use of a walker or cane during 6MWT were excluded.

**Fig. (1):**
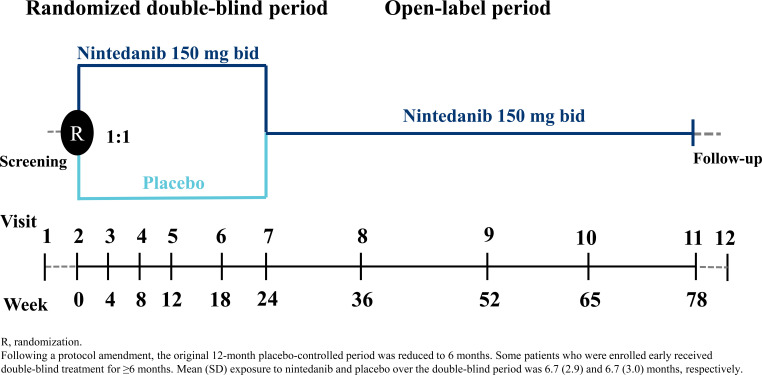
Trial design.

Recruitment began in December 2013. Originally patients were randomized 1:1 to receive nintedanib 150 mg bid or placebo double-blind for 12 months; however, this was amended to 6 months in a protocol amendment in October 2014 due to difficulties with enrolment following the US regulatory approval of nintedanib. As a result, the sample size of the study was reduced from 275 to approximately 150 patients, the primary endpoint was evaluated at month 6 rather than month 12, and all efficacy analyses were deemed exploratory. For some patients, the double-blind period was >6 months. The randomized treatment period was followed by an open-label period in which all patients received nintedanib (Fig. **1**).

Dose reductions to 100 mg bid and treatment interruptions were allowed to manage adverse events. Re-escalation to 150 mg bid was permitted once an adverse event had resolved. The trial was carried out in compliance with the protocol, and in accordance with the principles of the Declaration of Helsinki, the International Council on Harmonization Good Clinical Practice guideline and applicable regulatory requirements. An independent ethics committee or institutional review board at each participating center approved the trial protocol. All patients provided written informed consent.

### 2.2 Patients and Public Involvement

Patients were not involved in the design, recruitment to, or conduct of the study included in this analysis.

### 2.3 Endpoints

The primary endpoint was the relative change from baseline in QLF score (%) at month 6. Protocol-defined secondary endpoints were absolute, relative and categorical changes from baseline in FVC at month 6 (categorical changes: decline in FVC >10% predicted or 200 mL, change in FVC ≤10% predicted and ≤200 mL, or increase in FVC >10% predicted or 200 mL); absolute changes from baseline in 6MWD, St George’s Respiratory Questionnaire (SGRQ) total score [27] and University of California San Diego Shortness of Breath Questionnaire (UCSD-SOBQ) score [28] at month 6; time to all-cause mortality, respiratory-related mortality, respiratory-related hospitalization and investigator-reported acute exacerbation (defined in [25]) over 6 months; and relative change from baseline in QLF score (%) at month 12. Further endpoints were changes from baseline in velocity during 6MWT, oxygen saturation nadir (lowest SpO_2_ during 6MWT), delta desaturation (resting SpO_2_ minus lowest SpO_2_ during 6MWT) and the proportion of patients with oxygen saturation nadir ≤88% at month 6; absolute and relative changes from baseline in FVC (mL) at month 12; and absolute changes from baseline in 6MWD, SGRQ total score and UCSD-SOBQ score at month 12. In *post-hoc* analyses, we assessed correlations between changes in QLF score (% and mL) and changes in FVC (mL) at month 6.

Standardized non-contrast thin section volumetric chest HRCT was performed at screening (baseline) at months 6, 12, and 18. All scans were digitally transferred for central review. Generation of the QLF score (%) required lung segmentation, de-noising (normalization of the image), grid sampling, calculation of texture features, classification by support vector machine and calculation of the percentage of pulmonary fibrosis [15]. A more detailed explanation of how the QLF score is calculated and example HRCT scans illustrating a change in QLF score are provided in Appendix S1. The reproducibility of the QLF score had previously been established for the 4 by 4 pixel grid (*i.e*., 16 pixels) as 0.008% (±0.072) for the whole lung. On Bland Altman analysis, ±0.14% (*i.e*., 2 × SD) was the margin of variation due to measurement variation [29]. With regards to the repeatability coefficient, the variation due to measurement variation in the grid sampling was ±0.19% (*i.e*., 2.77 × SD) [30]. Assessment of QLF score in % was defined in the protocol. Absolute changes from baseline in QLF score, measured in mL, at months 6 and 12 were assessed *post-hoc*. QLF score in mL was calculated as the product of the segmented lung volume in mL and the % of pulmonary fibrosis. This allowed for the effect of lung volume, which could be influenced by different breath hold efforts, to be minimized.

Spirometry (FVC) was conducted at baseline and at months 3, 6, 9, 12, 15 and 18 using sponsor-provided spirometers and in compliance with ATS/ERS guidance [31]. A 6MWT was performed at baseline and at months 3, 6, 9, 12, 15 and 18. A practice 6MWT was conducted at screening to acquaint the patient with the procedure and determine the level of oxygen needed for subsequent tests. Investigators were provided with a strict protocol for conducting the 6MWT and a video for training their staff (available at: http://www.ussci comms.com/respiratory/lancaster/6mwt/).

Safety was assessed by clinical and laboratory evaluation and the recording of adverse events with onset after the first dose and up to 28 days after the last dose of study drug. Adverse events were coded according to the Medical Dictionary for Regulatory Activities (MedDRA) version 19.1.

### 2.4 Statistical Analyses

All analyses were exploratory and descriptive. Analyses were based on patients who received ≥1 dose of trial medication. Relative change from baseline in QLF score (%) at month 6 was analyzed using an analysis of covariance model, with fixed effects for treatment and baseline QLF score (%), in patients who had valid data for this endpoint. Change from baseline in QLF score (%) at month 12 was analyzed in patients who had valid QLF scores (%) at both months 6 and 12. Changes from baseline in QLF score (mL) at months 6 and 12 were analyzed in the same patients using an analysis of covariance model, with fixed effects for treatment and baseline QLF score (mL). Changes from baseline in FVC, 6MWD, SGRQ total score and UCSD-SOBQ score at months 6 and 12 were analyzed using a mixed model for repeated measures, with fixed effects for treatment, visit, treatment-by-visit, baseline value of the endpoint in question, baseline value of the endpoint in question-by-visit and random effect for patient. The model for change from baseline in FVC included sex, age and height as additional fixed effects. Analyses of time-to-event endpoints were not performed due to the small number of events. Correlations between changes in QLF score and changes in FVC at month 6 were assessed using Pearson (r) and Spearman correlation coefficients. Missing data were not imputed.

## 3 RESULTS

### 3.1 Patients

**Fig. (2):**
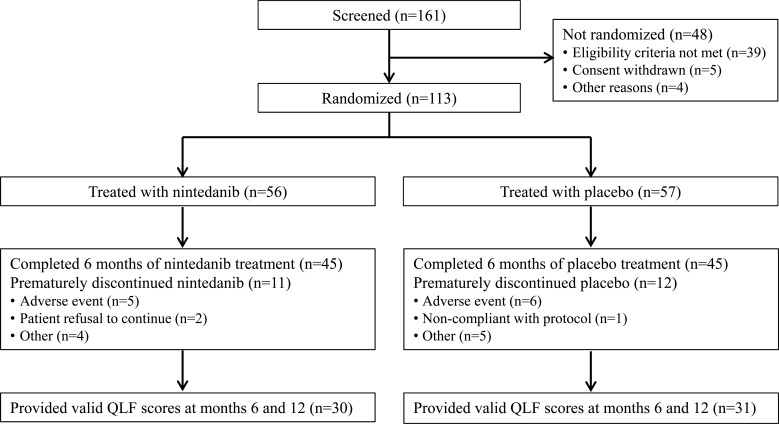
Patient disposition. QLF, quantitative lung fibrosis.

In total, 56 and 57 patients received ≥1 dose of nintedanib and placebo, respectively (Fig. **2**). There was a higher proportion of male patients in the nintedanib group than in the placebo group (80.4% *vs* 64.9%). At baseline, FVC, 6MWD and QLF score were similar between treatment groups (Table **1**). However, mean (SD) SGRQ and UCSD-SOBQ total scores were lower in the nintedanib group than in the placebo group (SGRQ, 35.8 [17.5] *vs* 44.4 [18.5]; UCSD-SOBQ, 25.4 [19.9] *vs* 42.3 [24.6]).

Over 6 months of randomized treatment, the proportion of patients who permanently discontinued study drug was similar between the nintedanib and placebo groups (19.6% and 21.1%, respectively). Dose reductions occurred in 12 patients (21.4%) treated with nintedanib and one (1.8%) treated with placebo. Treatment interruptions occurred in 13 patients (23.2%) treated with nintedanib and three (5.3%) treated with placebo. Mean (SD) exposure to nintedanib and placebo over the double-blind period was 6.7 (2.9) and 6.7 (3.0) months, respectively. Maximum exposure was 12.1 and 11.3 months, respectively.

### 3.2 QLF Scores

**Fig. (3):**
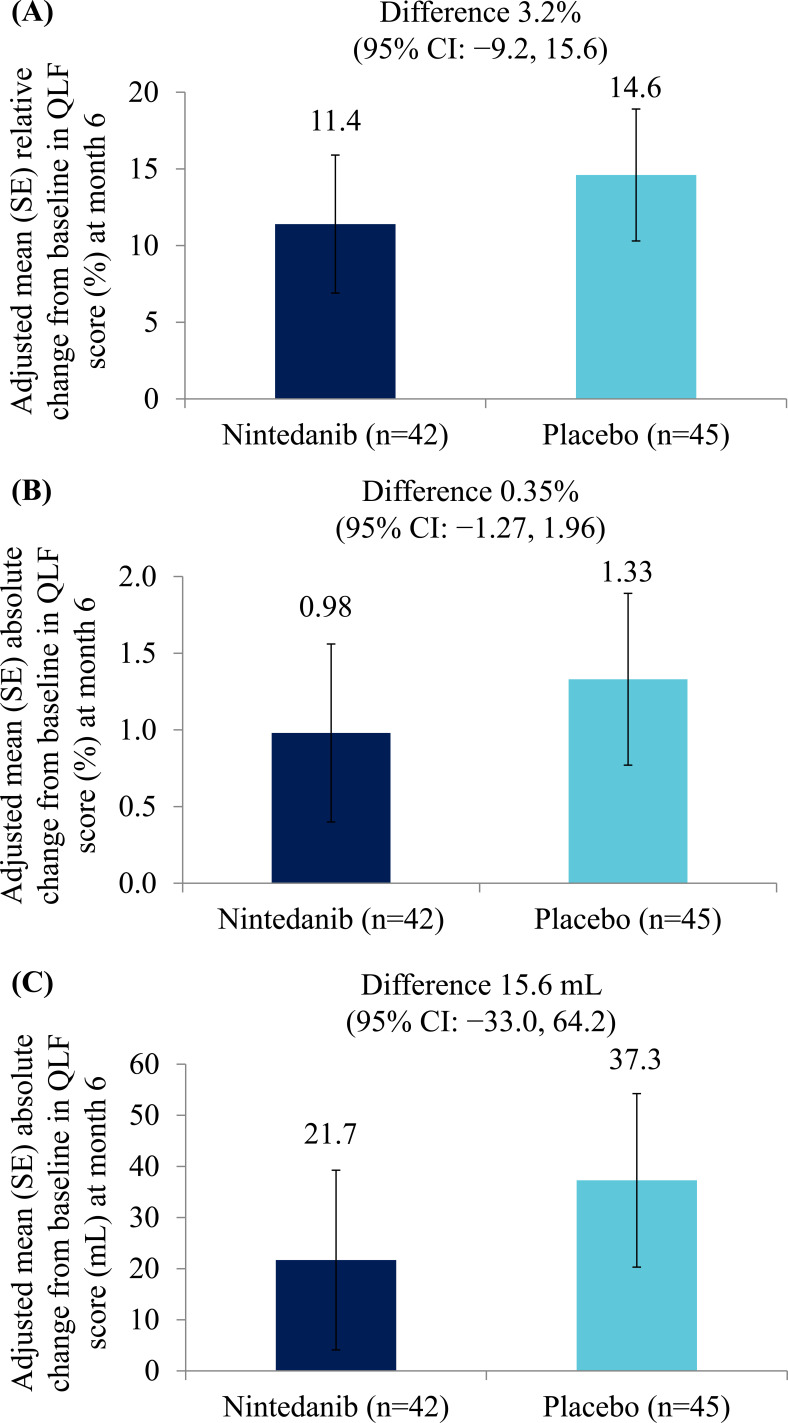
Relative (**A**) and absolute (**B**) changes from baseline in Quantitative Lung Fibrosis (QLF) score (%) at month 6; (**C**) absolute change from baseline in QLF score (mL) at month 6.

Adjusted mean (SE) relative changes from baseline in QLF score (%) at month 6 were 11.4 (4.5) % in the nintedanib group (n=42) and 14.6 (4.3) % in the placebo group (n=45) (difference of 3.2% [95% CI: −9.2, 15.6]) (Fig. **3A**). Adjusted mean (SE) absolute changes from baseline in QLF score at month 6 were 0.98 (0.58) % in the nintedanib group (n=42) and 1.33 (0.56) % in the placebo group (n=45) (difference of 0.35% [95% CI: −1.27, 1.96]) (Fig. **3B**). Adjusted mean (SE) absolute changes from baseline in QLF score (mL) at month 6 were 21.7 (17.6) mL in the nintedanib group (n=42) and 37.3 (17.0) mL in the placebo group (n=45) (difference of 15.6 mL [95% CI: −33.0, 64.2]) (Fig. **3C**). Changes from baseline in QLF score (% and mL) at month 12 are shown in Table **2**.

### 3.3 Forced Vital Capacity (FVC)

Adjusted mean (SE) absolute changes from baseline in FVC at month 6 were −14.2 (31.1) mL with nintedanib (n=54) and −83.2 (28.1) mL with placebo (n=54) (difference of 69.0 mL [95% CI: −8.7, 146.8]). Adjusted mean (SE) relative changes from baseline in FVC at month 6 were −0.7 (1.0) % in the nintedanib group (n=54) and −3.0 (1.0) % in the placebo group (n=54) (difference of 2.4% [95% CI: −0.29, 5.0]). A decline in FVC >10% predicted or >200 mL at month 6 was observed in 6.5% of patients in the nintedanib group and 23.9% in the placebo group. An increase in FVC >10% predicted or >200 mL at month 6 was observed in 17.4% of patients in the nintedanib group and 4.3% of patients in the placebo group. A change in FVC ≤10% predicted and ≤200 mL at month 6 was observed in 76.1% of patients in the nintedanib group and 71.7% of patients in the placebo group.

Adjusted mean (SE) absolute changes from baseline in FVC at month 12 were −30.9 (39.2) mL in patients who received nintedanib for 12 months (n=54) and −94.1 (37.1) mL in patients who received placebo for ≥6 months followed by open-label nintedanib (n=54) (difference of 63.2 mL [95% CI: −39.9, 166.3]). Adjusted mean (SE) relative changes from baseline in FVC at month 12 were −1.4 (1.4) % and −3.0 (1.3) % in these groups, respectively (difference of 1.7% [95% CI: −1.9, 5.3]).

### 3.4 Correlations between changes in QLF score and changes in FVC

Moderate negative correlations were observed between change in QLF score (%) at month 6 and change in FVC (mL) at month 6 (r^2^=0.34; Spearman = −0.62) (Figure **S1**) and between change in QLF score (mL) at month 6 and change in FVC (mL) at month 6 (r^2^=0.43; Spearman = −0.63) (Figure **S2**).

### 3.5 6-Minute Walk Test

6MWT outcomes at month 6 are summarized in Table **3**. Adjusted mean (SE) absolute changes from baseline in 6MWD at month 6 were 5 (11) m with nintedanib (n=55) and ‒13 (11) m with placebo (n=52) (difference of 18 m [95% CI: −14, 50]).

Adjusted mean (SE) absolute changes from baseline in 6MWD at month 12 were −15 (13) m in patients who received nintedanib for 12 months (n=55) and −11 (12) m in patients who received placebo for ≥6 months followed by open-label nintedanib (n=52) (difference of –4 m [95% CI: −39, 31]).

### 3.6 Health-Related Quality of Life

Results related to health-related quality of life are included in Appendix S2.

### 3.7 Hospitalizations, Acute Exacerbations and Deaths

Based on time to each event over 6 months, one patient in each group had an investigator-reported acute exacerbation; respiratory hospitalizations occurred in no patients in the nintedanib group and four patients (7.0%) in the placebo group; no patients died in the nintedanib group and three patients (5.3%) died in the placebo group.

### 3.8 Adverse Events

Adverse events are summarized in Table **4**. Over 6 months, diarrhea was reported in 38 patients (67.9%) treated with nintedanib and 18 patients (31.6%) treated with placebo. Adverse events leading to permanent discontinuation of trial medication occurred in five patients (8.9%) treated with nintedanib and three (5.3%) treated with placebo. Two patients (3.6%) in the nintedanib group and one (1.8%) in the placebo group had ALT and/or AST ≥3 × upper limit of normal.

Serious adverse events were reported in four patients (7.1%) in the nintedanib group and seven (12.3%) in the placebo group (Table **S1**). Based on adverse events classified based on MedDRA preferred term, no type of serious adverse event was reported in ≥1 patient treated with nintedanib. Adverse events leading to death were reported in one patient (1.8%) in the nintedanib group and four patients (7.0%) in the placebo group (Table **S2**).

## 4 DISCUSSION

This is the first trial to assess the effects of nintedanib on fibrotic changes evident on HRCT. Exploratory data suggest that compared with placebo, 6 months’ treatment with nintedanib was associated with a numerically smaller degree of fibrotic change in the lungs assessed by relative change in QLF score. A numerically smaller degree of fibrotic change was observed in patients treated with nintedanib for 12 months compared with placebo for at least 6 months followed by nintedanib until month 12. These data support the consideration of early treatment of IPF rather than delaying therapy. The finding of a reduced degree of fibrotic change in the lungs on HRCT is consistent with non-clinical research, which has shown that nintedanib, through inhibition of tyrosine kinase signaling pathways, interferes with processes involved in the progression of pulmonary fibrosis [32-34].

The absolute changes in QLF score (%) observed in this trial were largely consistent with those reported in other studies in patients with IPF [16, 19] and SSc-ILD [35, 36]. The Minimal Clinically Important Difference (MCID) in change in QLF score (%) in patients with IPF has not been established, but considering that the reproducibility of the QLF score is <0.2% [29], the absolute changes at months 6 and 12 of 0.9% and 1.4% in the nintedanib group and 1.3% and 2.2% in the placebo group support a positive impact of nintedanib on slowing the progression of fibrosis and the potential of the QLF score as a measure of treatment effect in an adequately powered study. In a cohort of 157 patients with IPF, a change in QLF score of 4% at month 6 was associated with an approximately 6-fold higher risk of a decline in FVC ≥10% predicted than a change in QLF score of <4%, adjusting for GAP stage [17].

The reduction in the decline in FVC with nintedanib versus placebo observed in this trial supported the findings on the QLF score and was consistent with the findings of the INPULSIS trials [25, 37]. At month 6, mean 6MWD and velocity had increased in the nintedanib group and decreased in the placebo group, with a between-group difference in 6MWD of 18 m. This suggests that nintedanib may improve exercise capacity in patients with IPF. The MCID for change in 6MWD in patients with IPF has been estimated as 22–45 m over approximately 1 year [7, 38, 39]; however, the use of different 6MWT protocols across studies limits comparisons [40].

Despite randomization, there were large imbalances between the treatment groups in SGRQ total score and UCSD-SOBQ score at baseline. No plausible reason could be identified for this. Given these imbalances, the observed changes in patient-reported outcomes at month 6 are challenging to interpret. However, the lack of difference between treatment groups in change in SGRQ total score is consistent with the INPULSIS trials [25]. The adverse event profile of nintedanib observed in this trial was consistent with previous trials, with diarrhea being the most frequent adverse event [24, 25]. No serious gastrointestinal adverse events were reported.

Our analyses have a number of limitations. Foremost, all efficacy endpoints were exploratory given the changes to the study protocol required after the US approval of nintedanib: following the reduction in sample size, the trial was not powered to show significant differences between treatment groups. Further, the protocol amendment resulted in patients receiving double-blind treatment for varying periods beyond month 6. Findings at month 6, and particularly at month 12, are biased by premature discontinuations that were more frequent in the placebo group.

## CONCLUSION

In this exploratory phase IIIb trial in patients with IPF, treatment with nintedanib was associated with a numerically smaller degree of fibrotic change in the lungs compared with placebo. These data, combined with the beneficial effects observed on FVC decline and 6MWD, support findings from previous trials showing that nintedanib reduces disease progression in patients with IPF.

## Figures and Tables

**Table 1:** Baseline characteristics.

-	**Nintedanib** **(n=56)**	**Placebo ** **(n=57)**
Male, n (%)	45 (80.4)	37 (64.9)
Age, years, mean (SD)	68.8 (7.6)	66.2 (9.4)
Race, n (%)	-	-
White	54 (96.4)	54 (94.7)
Asian	2 (3.6)	3 (5.3)
Time since diagnosis of IPF, years, mean (SD)	1.5 (1.4)	1.5 (1.4)
Smoking status, n (%)	-	-
Never	14 (25.0)	17 (29.8)
Former	41 (73.2)	40 (70.2)
Current	1 (1.8)	0 (0.0)
Body mass index, kg/m^2^, mean (SD)	29.4 (3.8)	30.3 (5.4)
FVC, mL, mean (SD)	2997 (831)	2921 (834)
FVC, % predicted, mean (SD)	78.0 (17.4)	78.1 (19.4)
DLco, % predicted, mean (SD)	53.6 (13.6)	52.5 (14.7)
QLF score, %, mean (SD)*	12.7 (7.9)	13.6 (8.1)
QLF score, mL, mean (SD)*	536 (316)	518 (262)
6MWD, m, mean (SD)	345 (141)	348 (146)
Oxygen saturation nadir during 6MWT, %, mean (SD)	89.6 (2.7)	89.7 (2.7)
Delta desaturation^†^, %, mean (SD)	6.2 (3.5)	6.3 (3.5)
Velocity during 6MWT, m/min, mean (SD)	57.6 (23.5)	58.0 (24.4)
SGRQ total score, mean (SD)	35.8 (17.5)	44.4 (18.5)
UCSD-SOBQ total score, mean (SD)^‡^	25.4 (19.9)	42.3 (24.6)

*n=42 and n=45 in the nintedanib and placebo groups, respectively. ^†^Resting SpO_2_ minus lowest SpO_2_ during 6MWT. ^‡^n=54 and n=55 in nintedanib and placebo groups, respectively. 6MWD, six-minute walk distance; 6MWT, six-minute walk test; DLco, diffusing capacity of the lung for carbon monoxide; FVC, forced vital capacity; IPF, idiopathic pulmonary fibrosis; QLF, quantitative lung fibrosis; SGRQ, St George’s Respiratory Questionnaire; UCSD-SOBQ, University of California San Diego Shortness of Breath Questionnaire.

**Table 2:** Changes from baseline in QLF score at month 12

**Endpoint**	**Nintedanib (n=30)**	Placebo* (n=31)
Adjusted mean (SE) relative changes from baseline in QLF score (%) at month 12, %	13.1 (4.5)	15.6 (4.5)
Difference (95% CI)	2.5 (−10.3, 15.3)	
Adjusted mean (SE) absolute changes from baseline in QLF score (%) at month 12, %	1.4 (0.6)	2.2 (0.5)
Difference (95% CI)	0.8 (−0.8, 2.3)	
Adjusted mean (SE) absolute changes from baseline in QLF score (mL) at month 12, mL	27.6 (18.0)	67.0 (17.7)
Difference (95% CI)	39.5 (−11.1, 90.0)	

*Patients received placebo for ≥6 months (mean: 6.7 months) followed by open-label nintedanib. QLF, quantitative lung fibrosis

**Table 3:** Changes from baseline in 6MWT endpoints at month 6.

**Endpoint**	**Nintedanib**	**Placebo**
6MWD	-	-
Number of patients	55	52
Mean (SE) change from baseline at month 6, m	5 (11)	‒13 (11)
Velocity during 6MWT	-	-
Number of patients	46	46
Mean (SD) change from baseline at month 6, m/min	1.2 (12.8)	−2.1 (13.7)
Oxygen saturation nadir during 6MWT	-	-
Number of patients	46	46
Mean (SD) change from baseline at month 6, %	1.2 (5.4)	1.4 (3.1)
Resting SpO_2_ minus lowest SpO_2_ during 6MWT (delta desaturation)	-	-
Number of patients	46	46
Mean (SD) change from baseline at month 6, %	1.1 (5.3)	1.4 (3.2)

6MWD, six-minute walk distance; 6MWT, six-minute walk test; SpO_2_, oxygen saturation.

**Table 4:** Most frequent adverse events reported over 6 months of randomized treatment.

-	**Nintedanib** **(n=56)**	**Placebo ** **(n=57)**
Any adverse event(s)	55 (98.2)	51 (89.5)
Most frequent adverse event(s)*	-	-
Diarrhea	38 (67.9)	18 (31.6)
Nausea	16 (28.6)	11 (19.3)
Fatigue	10 (17.9)	6 (10.5)
Decreased appetite	9 (16.1)	4 (7.0)
Vomiting	9 (16.1)	2 (3.5)
Headache	7 (12.5)	6 (10.5)
Cough	4 (7.1)	7 (12.3)
Any serious adverse event(s)^†^	4 (7.1)	7 (12.3)
Fatal adverse event	1 (1.8)	4 (7.0)
Any adverse event(s) leading to treatment discontinuation	5 (8.9)	3 (5.3)
Progression of IPF^‡^	0 (0.0)	3 (5.3)
Other	5 (8.9)	0 (0.0)

Data shown are n (%) of patients in whom ≥1 such adverse event was reported. *Adverse events reported in >12% of patients in either treatment group. ^†^Event that resulted in death, was immediately life-threatening, resulted in persistent or clinically significant disability or incapacity, required or prolonged hospitalization, was related to a congenital anomaly or birth defect, or was deemed serious for any other reason. ^‡^Corresponds to Medical Dictionary for Regulatory Activities term ‘IPF’, which included disease worsening and acute exacerbations of IPF. IPF, idiopathic pulmonary fibrosis.

## LIST OF ABBREVIATIONS

6MWD: = Six-Minute Walk Distance
6MWT: = Six-Minute Walk Test
ALT: = Alanine Transaminase
AST: = Aspartate Aminotransferase
DLco: = Diffusing Capacity of the Lung for Carbon Monoxide
FVC: = Forced vital capacity
HRCT: = High-resolution Computed Tomography
ILD: = Interstitial Lung Disease
IPF: = Idiopathic Pulmonary Fibrosis
MedDRA: = Medical Dictionary for Regulatory Activities
QLF: = Quantitative Lung Fibrosis
SGRQ: = St George’s Respiratory Questionnaire
UCSD-SOBQ: = University of California San Diego Shortness of Breath Questionnaire
