# Supplementary material for: Effects of Nintedanib on Quantitative Lung Fibrosis Score in Idiopathic Pulmonary Fibrosis
Source: Open Respir Med J. 2020 Sep 22;14:22–31. doi: 10.2174/1874306402014010022 (PMC7539538; doi:10.2174/1874306402014010022)
Supplement: Supplementary file 1 — Supplementary material is available on the publishers website along with the published article. [file TORMJ-14-22_Sp.pdf]

1874-3064/20

Send Orders for Reprints to reprints@benthamscience.net

The Open Respiratory Medicine Journal

Content list available at: https://openrespiratorymedicinejournal.com

Supplementary Material

Effects of Nintedanib on Quantitative Lung Fibrosis Score in Idiopathic Pulmonary Fibrosis

Lisa Lancaster[1,*], Jonathan Goldin[2], Matthias Trampisch[3], Grace Hyun Kim[2,4], Jonathan Ilowite[5], Lawrence Homik[6], David L. Hotchkin[7], Mitchell Kaye[8], Christopher J. Ryerson[9], Nesrin Mogulkoc[10] and Craig S Conoscenti[11]

[1]*Department of Medicine, Vanderbilt University Medical Center, Nashville, Tennessee, USA*
[2]*Department of Radiology, University of California, Los Angeles, California, USA*
[3]*Boehringer Ingelheim International GmbH, Ingelheim am Rhein, Germany*
[4]*Department of Biostatistics, University of California, Los Angeles, California, USA*
[5]*Division of Pulmonary and Critical Care Medicine, Department of Medicine, Hofstra North Shore-LIJ School of Medicine, New Hyde Park, New York*
[6]*Department of Respiratory Medicine and Bronchoscopy, Winnipeg Clinic, Winnipeg, Manitoba, Canada*
[7]*The Oregon Clinic, Division of Pulmonary, Critical Care & Sleep Medicine, Portland, Oregon, USA*
[8]*Department of Pulmonary Medicine, Minnesota Lung Center, Ltd., Minneapolis, Minnesota, USA*
[9]*Department of Medicine & Centre for Heart Lung Innovation, University of British Columbia, Vancouver, Canada*
[10]*Department of Pulmonology, Ege University Hospital, Bornova, Izmir, Turkey*
[11]*Boehringer Ingelheim Pharmaceuticals, Inc., Ridgefield, Connecticut, USA*

Article History	Received: March 31, 2020	Revised: June 17, 2020	Accepted: July 02, 2020

SUPPORTIVE/SUPPLEMENTARY MATERIAL

Appendix S1. Calculation of the Quantitative Lung Fibrosis (QLF) Score

To compute the QLF panel within a region of interest [1, 2]:

(1) Each pixel from a 4-by-4 grid within a segmented lung is sampled.

(2) Texture features from a denoised CT image for each sampled pixel are calculated.

(3) A previously built Support Vector Machine (SVM) classifier predicts reticulation of each pixel using the selected texture features. Features from denoised images are used to predict fibrotic reticulation or non-fibrotic reticulation, such as normal lung, ground glass, and honeycomb.

(4) The QLF score percentage (reticulation with architectural distortion) by lobes and lungs from the segmented regions is calculated using this formula:

$$QLF = \frac{\textit{Counts of pixels classified reticular pattern}}{\textit{Total counts of grid sample}}$$

(5) The QLF volume by lobes and lungs from the segmented regions is calculated:

$$\textit{QLF volume} = \textit{percent QLF} \times \textit{lung volume, where QLF is in range 0.0 to 1.0}$$

Example HRCT scans illustrating a change in QLF score (%) of 2.7

REFERENCES

[1] Kim HG, Tashkin DP, Clements PJ, et al. A computer-aided diagnosis system for quantitative scoring of extent of lung fibrosis in scleroderma patients. Clin Exp Rheumatol 2010;28(5 Suppl 62):S26–35.

[2] Kim HJ, Brown MS, Chong D, et al. Comparison of the quantitative CT imaging biomarkers of idiopathic pulmonary fibrosis at baseline and early change with an interval of 7 months. Acad Radiol 2015;22:70–80.

Appendix S2. Health-Related Quality of Life

At baseline, mean (SD) SGRQ total scores were 35.8 (17.5) and 44.4 (18.5) in the nintedanib and placebo groups, respectively. Adjusted mean (SE) absolute changes from baseline in SGRQ total score at month 6 were −2.24 (1.68) in the nintedanib group (n=55) and −2.19 (1.70) in the placebo group (n=53) (difference −0.05 [95% CI: −4.89, 4.79]. At baseline, mean (SD) UCSD-SOBQ scores were 25.4 (19.9) and 42.3 (24.6) in the nintedanib and placebo groups, respectively. Adjusted mean (SE) absolute changes from baseline in UCSD-SOBQ score at month 6 were 3.42 (2.16) in the nintedanib group (n=53) and −1.46 (2.19) in the placebo group (n=50) (difference of 4.89 [95% CI: −1.47, 11.25]).

DOI: 10.2174/1874306402014010022, 2020, *14*, i-iv

Adjusted mean (SE) absolute changes from baseline in SGRQ total score at month 12 were −3.12 (2.14) in patients who received nintedanib for 12 months (n=55) and −1.13 (2.12) in patients who received placebo for ≥6 months followed by open-label nintedanib (n=53) (difference of −1.99 [95% CI: −8.17, 4.19]). Adjusted mean (SE) absolute changes from baseline in UCSD-SOBQ score at month 12 were 4.43 (2.45) in patients who received nintedanib for 12 months (n=53) and 1.29 (2.38) in patients who received placebo followed by nintedanib (n=50) (difference of 3.15 [95% CI: −4.01, 10.31]).

Table S1. Serious adverse events reported (irrespective of causality) over 6 months of randomized treatment*.

	**Nintedanib (n=56)**	**Placebo (n=57)**
Any serious Adverse Events	4 (7.1)	7 (12.3)
Progression of IPF*	0 (0.0)	3 (5.3)
Pneumonia	0 (0.0)	2 (3.5)
Hypoxia	1 (1.8)	0 (0.0)
Intestinal infarction	1 (1.8)	0 (0.0)
Lyme disease	1 (1.8)	0 (0.0)
Spinal column stenosis	1 (1.8)	0 (0.0)
Spinal osteoarthritis	1 (1.8)	0 (0.0)
Completed suicide	0 (0.0)	1 (1.8)
Delirium	0 (0.0)	1 (1.8)
Flank pain	0 (0.0)	1 (1.8)
Haemoptysis	0 (0.0)	1 (1.8)
Musculoskeletal pain	0 (0.0)	1 (1.8)
Osteoarthritis	0 (0.0)	1 (1.8)
Pleural effusion	0 (0.0)	1 (1.8)
Respiratory failure	0 (0.0)	1 (1.8)

*Adverse events with onset after the first dose and up to 28 days after the last dose of study drug. Data shown are n (%) of patients in whom ≥1 such event was reported. A serious adverse event was defined as an adverse event that resulted in death, was immediately life-threatening, resulted in persistent or clinically significant disability or incapacity, required or prolonged hospitalization, was related to a congenital anomaly or birth defect, or was deemed serious for any other reason. *Corresponds to Medical Dictionary for Regulatory Activities term ‘IPF’, which included disease worsening and acute exacerbations of IPF. IPF, idiopathic pulmonary fibrosis.

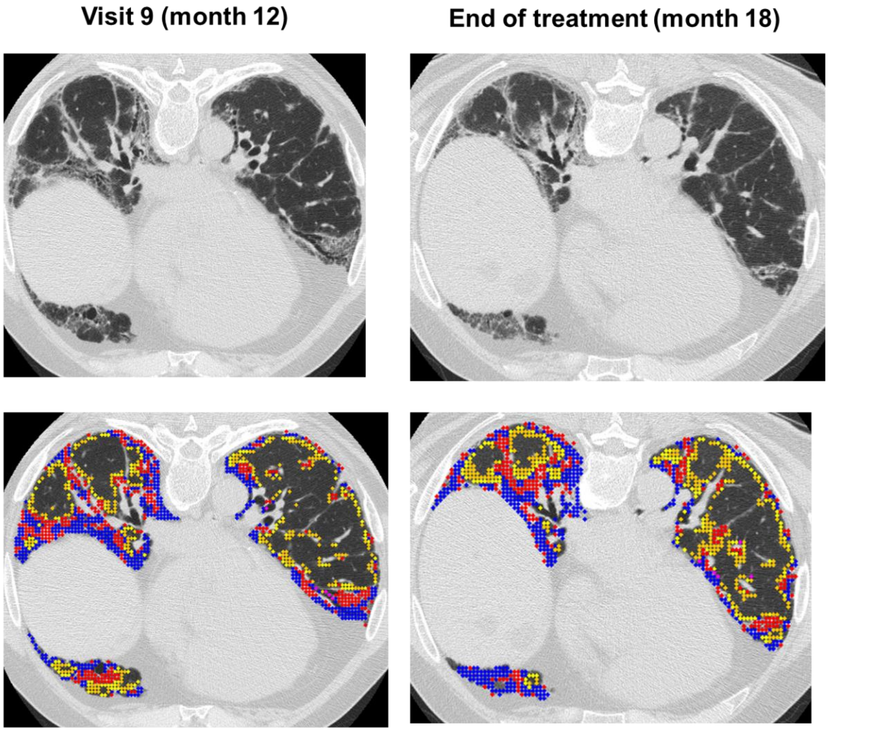

QLF (red+blue) score in whole lung (%) = 17.6% at Visit 9; 20.3% at end of treatment
QLF (red+blue) score in whole lung (mL) = 706 mL at Visit 9; 714 mL at end of treatment

Fig. S1 contd.....

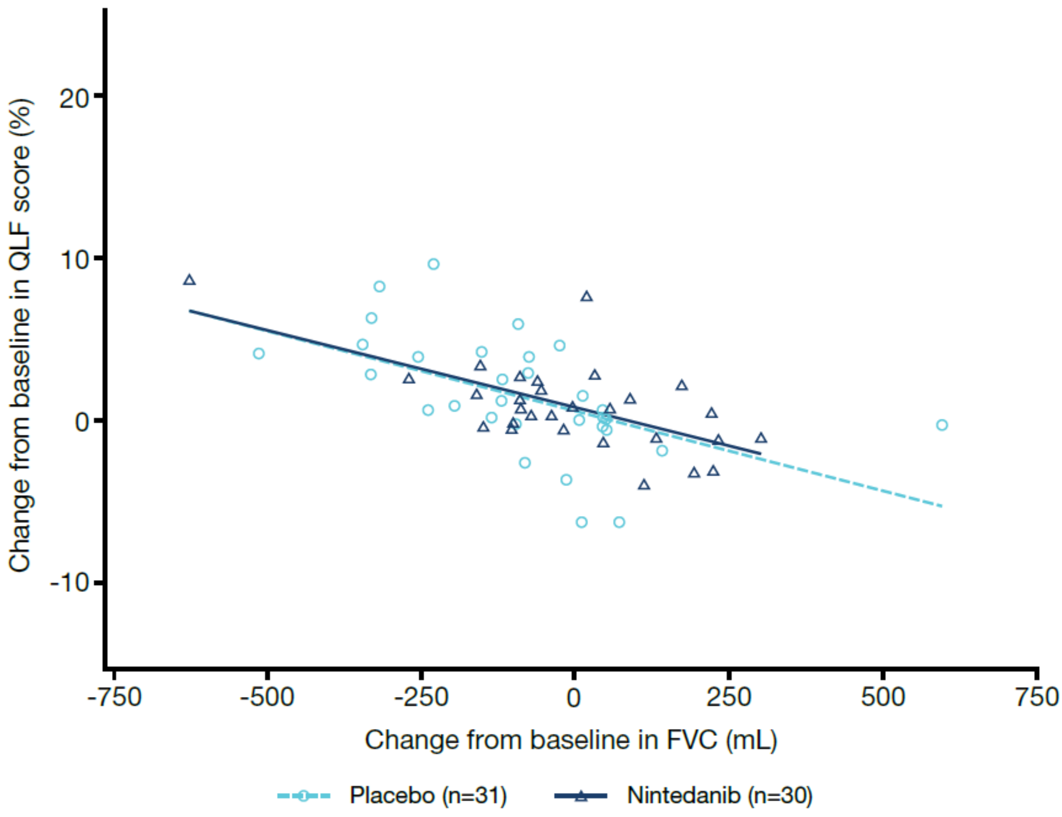

Fig. (S1). Correlation between change from baseline in QLF score (%) and change from baseline in FVC (mL) at month 6

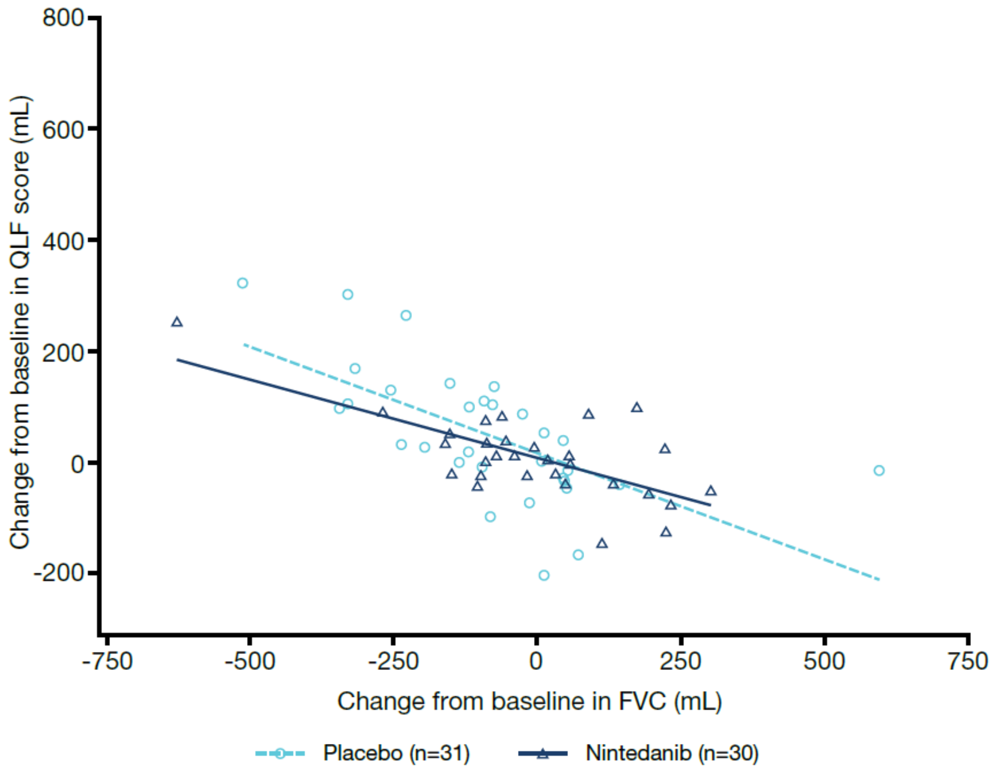

Fig. (S2). Correlation between change from baseline in QLF score (mL) and change from baseline in FVC (mL) at month 6

Table S2. Adverse events leading to death reported (irrespective of causality) over 6 months of randomized treatment*

	Nintedanib (n=56)	Placebo (n=57)
Any fatal Adverse Events	1 (1.8)	4 (7.0)
Intestinal infarction	1 (1.8)	0
Progression of IPF*	0	1 (1.8)
Pleural effusion	0	1 (1.8)
Respiratory failure	0	1 (1.8)
Suicide	0	1 (1.8)

*Adverse events with onset after the first dose and up to 28 days after the last dose of study drug. Data shown are n (%) of patients. *Corresponds to Medical Dictionary for Regulatory Activities term ‘IPF’, which included disease worsening and acute exacerbations of IPF. IPF, idiopathic pulmonary fibrosis.

© 2020 Lancaster *etal*.

This is an open access article distributed under the terms of the Creative Commons Attribution 4.0 International Public License (CC-BY 4.0), a copy of which is available at: (https://creativecommons.org/licenses/by/4.0/legalcode). This license permits unrestricted use, distribution, and reproduction in any medium, provided the original author and source are credited.
